# Feature selection using Haar wavelet power spectrum

**DOI:** 10.1186/1471-2105-7-432

**Published:** 2006-10-05

**Authors:** Prabakaran Subramani, Rajendra Sahu, Shekhar Verma

**Affiliations:** 1 ABV-Indian Institute of Information Technology and Management, Gwalior, India

## Abstract

**Background:**

Feature selection is an approach to overcome the 'curse of dimensionality' in complex researches like disease classification using microarrays. Statistical methods are utilized more in this domain. Most of them do not fit for a wide range of datasets. The transform oriented signal processing domains are not probed much when other fields like image and video processing utilize them well. Wavelets, one of such techniques, have the potential to be utilized in feature selection method. The aim of this paper is to assess the capability of Haar wavelet power spectrum in the problem of clustering and gene selection based on expression data in the context of disease classification and to propose a method based on Haar wavelet power spectrum.

**Results:**

Haar wavelet power spectra of genes were analysed and it was observed to be different in different diagnostic categories. This difference in trend and magnitude of the spectrum may be utilized in gene selection. Most of the genes selected by earlier complex methods were selected by the very simple present method. Each earlier works proved only few genes are quite enough to approach the classification problem [1]. Hence the present method may be tried in conjunction with other classification methods. The technique was applied without removing the noise in data to validate the robustness of the method against the noise or outliers in the data. No special softwares or complex implementation is needed. The qualities of the genes selected by the present method were analysed through their gene expression data. Most of them were observed to be related to solve the classification issue since they were dominant in the diagnostic category of the dataset for which they were selected as features.

**Conclusion:**

In the present paper, the problem of feature selection of microarray gene expression data was considered. We analyzed the wavelet power spectrum of genes and proposed a clustering and feature selection method useful for classification based on Haar wavelet power spectrum. Application of this technique in this area is novel, simple, and faster than other methods, fit for a wide range of data types. The results are encouraging and throw light into the possibility of using this technique for problem domains like disease classification, gene network identification and personalized drug design.

## Background

Modern technologies like microarrays facilitate the study of expression levels of thousands of genes simultaneously. This study is useful to determine whether these genes are active, hyperactive or inactive in various tissues. The vast amount of microarray data is so important for the applications like disease classification and identifying the genetic networks. Solutions for complex problems like identification of cancer types and their subtypes need more accuracy for utilizing them in treating this disease and in preparing more effective therapeutic solution like individual drug design. So, it is important and necessary to select only genes containing the expression data contributing to the problem domain and to filter irrelevant data to increase the performance of the methods used. Feature selection is the problem of identifying such genes or features [2]. That is, this can be used to identify the important genes with significant information content when the problem is poorly structured. This improves the generalization performance and inference of classification models [3] by overcoming the 'curse of dimensionality'. One important problem with feature selection methods is that both problem relevance and biological relevance of the features selected may not be achieved completely. Also, most of the feature selection methods do not fit for the wide range of datasets. They are coupled with a particular classification method and time consuming. Statistical methods are in use in this domain for a long time. But, extensive preprocessing and lesser consensus among them are major problems with them. Transform oriented signal processing methods are simpler and may provide an alternative platform to the statistical methods. They have been successfully utilized in many domains like image processing. But, they have not been much utilized in the field of bioinformatics. The key advantage of these transform oriented methods is their power of capturing some inherent properties of the data. The aim of this paper is to analyse the capabilities of Haar wavelet power spectrum in selecting informative features in microarray data on the basis of the inherent properties captured by them. The present work utilizes some earlier works in feature selection for illustration and analyses the comparability of wavelet strategy with those of earlier works.

### Feature selection

Feature selection can be approached in three ways. First, we may handle feature selection method independently irrespective of the further applications utilizing these features. That is, the features selected may be used for any classifier algorithms. This approach of feature selection is called a filter method. Second, features may be selected for a specific classifier algorithm. In this approach called a 'wrapper method' [4], the qualities or accuracies of all possible subsets are analyzed to select the optimal one to the specific classification algorithm. Finally, feature selection and classifier design may be accomplished together. This strategy is found in embedded methods. Embedded methods are incorporated into the learning procedure, and hence are dependent on the classification model.

Systematizations and surveys on feature selection algorithms have been presented in a variety of review articles like Blum and Langley [5], Kohavi and John [4] and Guyon [6]. So far, a number of variable (or gene) selection methods like the support vector machine method [5], the genetic algorithm [7], the perceptron method [8], Bayesian variable selection [9-12], and the voting technique [13], mutual information-based gene and feature selection method [1], entropy based feature selection [14] and many artificial intelligent techniques like hill climbing, best first search [15], simulated annealing [16], backward elimination [17], forward selection and their combinations have been proposed. Specific to filter approach, Kira and Rendell's Relief algorithm [18] which selects features based on a threshold of weights assigned to each feature is a good example but it was tested on small set of features.

In case of high dimensional datasets containing thousands of genes, filters are preferred to wrappers due to their independency over the models [19,20]. Xiang et al [14] devised a hybrid of filter and wrapper approaches and tested it on high dimensional gene expression data with 72 samples and 7129 features. Another such work on high dimensional gene expression data was done by Golub et al [13] on the same dataset using correlation measures. Califano et al [21] also worked on a high dimensional dataset of 6817 genes using a supervised learning algorithm. All these works revealed the fact that the result was better while using selected features instead of the whole data set.

Most commonly used filters are based on information-theoretic or statistical principles. Score based feature selection methods are popular among filters using statistical principles. These methods calculate statistical scores on the gene expression data. They sort genes according the scores assigned and filter them by applying some threshold. χ 2-score, t-test metrics[22], Wilcoxon rank sum test[22], correlation co efficient [23] and B-scatter score are some prominent examples. Some other strategies used in feature selection through ranking are SNR based ranking used in Shipp's approach [24] and sensitivity analysis based ranking used in Mean square classifier [25]. The strategy of selecting features using sensitivity analysis is to rank a feature according to the change in the value of an objective function caused by the removal of that feature from the dataset. SNR method is more capable of detecting and ranking a smaller number of significant variables. Apart from ranking methods, several other approaches like Relief [18,26], gini-index [27], relevance, average absolute weight of evidence [28] and bi-normal separation [29] are also in use.

Most of the methods of feature selection are complex and consume more time to converge. Many of them do not fit for all data types in addition that they require more samples. No consensus among various statistical methods is achieved to use them. The selection of a statistical method for a dataset is a hit and run approach. So, a more generic method which can cope up with a variety of data is in dire need. Further, a very few model independent approaches for feature selection are available since most of the methods of feature selection are coupled with classification. In this paper, we analyse the capability of wavelet power spectrum in feature selection and we propose a method of feature selection based on Haar wavelet power spectrum. This method is found fit for a wide range of data sets and also works with smaller number of samples. It can be used in conjunction with other classification methods. The algorithm is very simple and requires comparatively less time to be executed. The method is a model independent approach, a filter feature selection method, based on the Haar wavelet power spectrum of the microarray data. Unlike most of the other methods, it is relatively a very simple algorithm. We observed that the features selected by our method can be used in conjunction with more classification algorithms.

### Wavelet and its power spectrum

**Figure 1:**
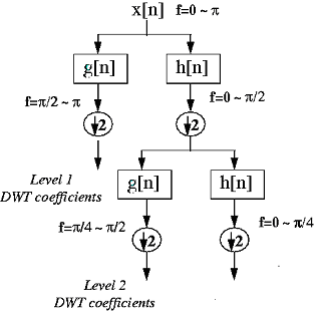
A two level DWT for N data. The number of data is halved after every filtering and down sampling operation. A wavelet transform is applied on output of low pass filter (h [n]) (approximation coefficients) recursively keeping the output coefficients of each high pass filtering operation (g [n]) (detailed coefficients) at each stage. The wavelet transform of a data at any level *i *of decomposition consists of approximation coefficients only at *i*th level and all detailed coefficients up to *i*^th ^level.

Wavelets are a family of basis functions that can be used to approximate other functions by expansion in orthonormal series. They combine such powerful properties as orthonormality, compact support, varying degrees of smoothness, localization both in time or space and scale (frequency), and fast implementation. One of the key advantages of wavelets is their ability to spatially adapt to features of a function such as discontinuities and varying frequency behaviour. A wavelet transform is a lossless linear transformation of a signal or data into coefficients on a basis of wavelet functions [30]. Performing the discrete wavelet transform (DWT) of a signal *x *is done by passing it through low pass filters (scaling functions) and high pass filters simultaneously. Down-sampling or decimation by a factor 2 is performed after each pass through filters. Decimation by 2 means removing every alternative coefficient in the function is performed after each pass through filters. Figure 1 depicts a two level wavelet transform.

Mathematically, the wavelet transform of a function x [k] can be represented by the following formula:

yhigh(n)=∑k=−∞∞x[k]⋅g[2⋅n−k]ylow(n)=∑k=−∞∞x[k]⋅h[2⋅n−k]
 MathType@MTEF@5@5@+=feaafiart1ev1aaatCvAUfKttLearuWrP9MDH5MBPbIqV92AaeXatLxBI9gBaebbnrfifHhDYfgasaacH8akY=wiFfYdH8Gipec8Eeeu0xXdbba9frFj0=OqFfea0dXdd9vqai=hGuQ8kuc9pgc9s8qqaq=dirpe0xb9q8qiLsFr0=vr0=vr0dc8meaabaqaciaacaGaaeqabaqabeGadaaakeaafaqabeGabaaabaGaemyEaK3aaSbaaSqaaiabdIgaOjabdMgaPjabdEgaNjabdIgaObqabaGccqGGOaakcqWGUbGBcqGGPaqkcqGH9aqpdaaeWbqaaiabdIha4jabcUfaBjabdUgaRjabc2faDjabgwSixlabdEgaNjabcUfaBjabikdaYiabgwSixlabd6gaUjabgkHiTiabdUgaRjabc2faDbWcbaGaem4AaSMaeyypa0JaeyOeI0IaeyOhIukabaGaeyOhIukaniabggHiLdaakeaacqWG5bqEdaWgaaWcbaGaemiBaWMaem4Ba8Maem4DaChabeaakiabcIcaOiabd6gaUjabcMcaPiabg2da9maaqahabaGaemiEaGNaei4waSLaem4AaSMaeiyxa0LaeyyXICTaemiAaGMaei4waSLaeGOmaiJaeyyXICTaemOBa4MaeyOeI0Iaem4AaSMaeiyxa0faleaacqWGRbWAcqGH9aqpcqGHsislcqGHEisPaeaacqGHEisPa0GaeyyeIuoaaaaaaa@77BC@

where *y*_*low *_(*n*) and *y*_*high *_(*n*) are responses from low and high pass filters respectively. In matrix form, wt = [WX^T^]^T ^where W = [L;H] where L and H are impulse responses of low pass and high pass filters and wt is wavelet transform of the one dimensional input signal X. The two filters used at each stage of decomposition must be related to each other by g [*l*-1-*n*] = (-1)^*n*^·*h*[*n*] where g and h are the impulse responses of the two filters, *l *is the filter length in number of points, *n *is the order of the data points and *l *is such that 0 ≤ *n *<*l*. For example, there are two data points for each filter of Haar wavelet with *n *= 0, 1. These filters are known as quadrature mirror filters. A wavelet transform of a data after *i *level of decompositions contains the approximation coefficients at *i*^th ^level and all detailed coefficients up to *i*^th ^level. The detailed coefficients at different levels incorporate the variations in information at those levels. Level of decomposition is also termed as band.

A number of wavelet families like symlet, coiflet, daubechies and biorthogonal wavelets are already in use. They vary in various basic properties of wavelets like compactness. Among them, Haar wavelets belonging to daubechies wavelet family are most commonly used wavelets in database literature because they are easy to comprehend and fast to compute [3]. Haar transform can be viewed as a series of averaging and differentiating operations on a discrete function. The impulse response for high pass filter is given by [1/2
 MathType@MTEF@5@5@+=feaafiart1ev1aaatCvAUfKttLearuWrP9MDH5MBPbIqV92AaeXatLxBI9gBaebbnrfifHhDYfgasaacH8akY=wiFfYdH8Gipec8Eeeu0xXdbba9frFj0=OqFfea0dXdd9vqai=hGuQ8kuc9pgc9s8qqaq=dirpe0xb9q8qiLsFr0=vr0=vr0dc8meaabaqaciaacaGaaeqabaqabeGadaaakeaadaGcaaqaaiabikdaYaWcbeaaaaa@2DB9@, -1/2
 MathType@MTEF@5@5@+=feaafiart1ev1aaatCvAUfKttLearuWrP9MDH5MBPbIqV92AaeXatLxBI9gBaebbnrfifHhDYfgasaacH8akY=wiFfYdH8Gipec8Eeeu0xXdbba9frFj0=OqFfea0dXdd9vqai=hGuQ8kuc9pgc9s8qqaq=dirpe0xb9q8qiLsFr0=vr0=vr0dc8meaabaqaciaacaGaaeqabaqabeGadaaakeaadaGcaaqaaiabikdaYaWcbeaaaaa@2DB9@] and for low pass filter, the impulse response is [1/2
 MathType@MTEF@5@5@+=feaafiart1ev1aaatCvAUfKttLearuWrP9MDH5MBPbIqV92AaeXatLxBI9gBaebbnrfifHhDYfgasaacH8akY=wiFfYdH8Gipec8Eeeu0xXdbba9frFj0=OqFfea0dXdd9vqai=hGuQ8kuc9pgc9s8qqaq=dirpe0xb9q8qiLsFr0=vr0=vr0dc8meaabaqaciaacaGaaeqabaqabeGadaaakeaadaGcaaqaaiabikdaYaWcbeaaaaa@2DB9@,1/2
 MathType@MTEF@5@5@+=feaafiart1ev1aaatCvAUfKttLearuWrP9MDH5MBPbIqV92AaeXatLxBI9gBaebbnrfifHhDYfgasaacH8akY=wiFfYdH8Gipec8Eeeu0xXdbba9frFj0=OqFfea0dXdd9vqai=hGuQ8kuc9pgc9s8qqaq=dirpe0xb9q8qiLsFr0=vr0=vr0dc8meaabaqaciaacaGaaeqabaqabeGadaaakeaadaGcaaqaaiabikdaYaWcbeaaaaa@2DB9@]. That is, the minimum number of elements in input data should be 2. The input data should always contain the number of elements 2^n ^where n is an integer. In matrix form, the Haar wavelet filter can be expressed as

[121212−12]
 MathType@MTEF@5@5@+=feaafiart1ev1aaatCvAUfKttLearuWrP9MDH5MBPbIqV92AaeXatLxBI9gBaebbnrfifHhDYfgasaacH8akY=wiFfYdH8Gipec8Eeeu0xXdbba9frFj0=OqFfea0dXdd9vqai=hGuQ8kuc9pgc9s8qqaq=dirpe0xb9q8qiLsFr0=vr0=vr0dc8meaabaqaciaacaGaaeqabaqabeGadaaakeaadaWadiqaauaabeqaciaaaeaadaWcaaqaaiabigdaXaqaamaakaaabaGaeGOmaidaleqaaaaaaOqaamaalaaabaGaeGymaedabaWaaOaaaeaacqaIYaGmaSqabaaaaaGcbaWaaSaaaeaacqaIXaqmaeaadaGcaaqaaiabikdaYaWcbeaaaaaakeaacqGHsisldaWcaaqaaiabigdaXaqaamaakaaabaGaeGOmaidaleqaaaaaaaaakiaawUfacaGLDbaaaaa@37F9@

It can be easily examined that both the low pass and high pass filters of Haar wavelet are quadratic in nature using the discussion presented in the previous paragraph. For a data having more than two elements, the Haar wavelet matrix of can be constructed by diagonally repeating these basic filters to form a matrix of the size of input data. Upper part of the matrix is created by repeating impulse responses of low pass filter diagonally and lower part of the matrix is created by repeating impulse responses of high pass filter diagonally. From Figure 1, it is evident that the size of the data points to be used for wavelet transform in a level is equal to half of the data points used in the previous level. Accordingly, the size of the Haar wavelet matrix also reduced. For example, if we use a signal of four data points, the size of the Haar wavelet matrix will be 4 × 4 in the first step of wavelet transform. From Figure 1, it is evident that the number of data points to be used for the second step of wavelet transform is 2. These are the output of low pass filtering operation as shown in Figure 1. So, the Haar wavelet matrix to be used is of the size 2 × 2. More details of wavelets may be referred at [31-33].

The minimum number of data points in an input signal should be 2 in the case of Haar wavelet and the number of data points needed for n times decomposition is 2^n^. If the number of input data points is less than this required number, 2^n^, zeros may be padded (appended) at the right end of the input data to compensate the required number. In the present work, the number of data points refers to the number of samples which is equal to the number of columns present in the microarray data matrix. That is, the expression of a gene in a sample is considered as a data point of a one dimensional signal X. Accordingly, the columns of the microarray data matrix were prepared so as to be amenable for satisfying the required number criterion. In some experiments, a reduced number of the columns of the microarray data matrix equal to the nearest power of 2 were randomly selected and used. Since we use the strategy of finding the average value of wavelet power spectrum for each gene per sample, in the present work, the choice of columns selected for replication or reduction is immaterial. We used a random selection of the columns for the purpose of reduction and replication for data input preparation. It was observed that such a random selection of columns did not affect much the robustness and the accuracy of the present method used. In the present work, expression data of each gene across various tissue samples or various experiments is modeled to a one dimensional signal. Therefore, the entire microarray data is modeled to a group of M number of one-dimensional signals where M is the total number of genes present in the gene microarray data. More mathematical details of wavelets may be referred at [31-33].

Local wavelet power spectrum at a particular decomposition level is calculated by summing up the squares of wavelet coefficients at that level [11]. For a set of wavelet coefficients C_j,k_, where j is level of decomposition and k is the order of the coefficient, the wavelet power spectrum is given below.

spectrum[j]=∑k=02j−1Cj,k2
 MathType@MTEF@5@5@+=feaafiart1ev1aaatCvAUfKttLearuWrP9MDH5MBPbIqV92AaeXatLxBI9gBaebbnrfifHhDYfgasaacH8akY=wiFfYdH8Gipec8Eeeu0xXdbba9frFj0=OqFfea0dXdd9vqai=hGuQ8kuc9pgc9s8qqaq=dirpe0xb9q8qiLsFr0=vr0=vr0dc8meaabaqaciaacaGaaeqabaqabeGadaaakeaacqqGZbWCcqqGWbaCcqqGLbqzcqqGJbWycqqG0baDcqqGYbGCcqqG1bqDcqqGTbqBcqGGBbWwcqqGQbGAcqGGDbqxcqGH9aqpdaaeWbqaaiabboeadnaaDaaaleaacqqGQbGAcqGGSaalcqqGRbWAaeaacqaIYaGmaaaabaGaee4AaSMaeyypa0JaeGimaadabaGaeGOmaiZaaWbaaWqabeaacqqGQbGAaaWccqGHsislcqaIXaqma0GaeyyeIuoaaaa@4C5A@

If there are N elements in an array, there will be log_2_(N) coefficient bands or levels of decomposition for Haar wavelet. That is, the power spectrum can be referred as a graphical representation of cumulative information variation at each scale of decomposition. Global wavelet power spectrum [34] is the average of such local power spectra.

## Results and discussion

Our proposed algorithm for feature selection has been applied on various datasets and top genes are reported here. In all these experiments, we have used Haar wavelets since the number of minimum features for wavelet transformation at lowest level is smaller than that required by the other wavelets. We applied our method on three datasets namely Golub dataset, Hedenfalk breast cancer dataset and Khan SRBCT dataset. All experiments were carried out without filtering any data to validate the robustness of the method against the noise or outliers in the data.

### SRBCT dataset

First, we focus on feature selection for the small, round blue cell tumors (SRBCT) of childhood. The dataset of SRBCT used for experimentation here is available at [35]. This dataset is composed of 2308 genes and 63 samples from four cancers which includes Neuroblastoma (NB) (12 samples), Rhabdomyosacoma (RMS) (23 samples), Burkitt Lymphomas (BL) (8 samples) and Ewing's family of tumors (EWS) (20 samples). Originally, Khan et al [35] classified this dataset using artificial neural networks on gene expression profiles. The feature selection and classification using this dataset has also been performed by Zhou et al using Gibb's sampler and SMC [36]. Khan et al [35] selected a list of 96 discriminating genes pertaining to classification. This list included some genes being identified important to two classes out of four classes and some genes which were not categorized for any class. Our method has identified some of them important for one of the four classes. It has selected almost all these features with comparatively simpler calculations. Also, we used only 32 samples out of 63 sample set. First four samples from each diagnostic category have been selected to form this group of 32 samples. It exhibits the possibility of using our methods for datasets with a lesser number of samples. Most of the top ranked genes listed in the present work have been used in classification of the dataset in earlier works [36,35].

A list of top genes selected by our method has been listed in Table 1. Genes with index IDs 1319, 1645, 1954, 1831, 2303, 1980, 373 and 1626 were also reported to be differentially expressed in EWS in Khan et al's work [35]. Gene 851 was not allocated to any class in [35]. Most of the other genes like gene 951 reported to be discriminating EWS have been selected as important genes for EWS but with a lower ranking. The rank of Gene 951 was 59 in our work and its RPV was 59.36. Gene 1200 was not selected in Khan's [35] work but it was ranked fourth in our work. Of all genes selected for EWS, neural-specific genes [37,38] like TUBB5 (Gene 373), ANXA1 (Gene 1831), and NOE1 (Gene 1645) lend more credence to the proposed neural histogenesis of EWS [39]. Most of the top ranked genes are dominant in EWS category in comparison with their expression in other classes. This implies that most of the top ranked genes in Table 1 are highly related to the classification of EWS from other categories. When tested with Golub's algorithm [13], first 23 samples of SRBCT dataset except sample number 12 were categorized as EWS and remaining 43 samples were categorized as non EWS samples.

**Table 1:** Differentially expressed genes selected for classifying EWS diagnostic category of SRBCT data (RPV – Relative percentage variation).

**Rank**	**Index no.**	**Clone ID**	**RPV (%)**
1	1319	866702	99.52
2	1645	52076	97.92
3	1954	814260	97.91
4	1200	838856	96.45
5	696	753587	95.63
6	1140	824922	92.71
7	1070	1475730	91.06
8	851	563673	89.27
9	404	1422723	88.28
10	1831	208718	87.64
16	1980	841641	83.46
19	373	291756	81.31
20	1626	811000	81.22

A list of strongest genes selected for classifying BL versus others using our method has been reported in Table 2. In the SRBCT dataset, the samples of BL category spans from sample number 24 to sample number 31. Among them, Genes 836 and 1158 were reported to be differentially expressed in BL in Khan's original work [35]. Genes with indices 1916, 783, 846, 1735,335,1884,2230,1915, 85 and 276 were also selected in [35] but not assigned to a particular class. Of six differentially expressed genes in BL and some other classes, remaining four genes except genes 836 and 1158 were selected for other classes. So, our method selects proper genes effectively with simple algorithm and calculations. Most of the top ranked genes are dominant in BL category in comparison with their expression in other classes (See additional file1). This implies that most of the top ranked genes in Table 2 are highly related to the classification of BL from other categories. Expression levels of these genes show that they can be classified using Golub's classification algorithm [13] since they are highly correlated to the "idealized expression pattern" [13].

**Table 2:** A list of top ranked genes selected by using relative percentage variation of gene expression profiles between BL versus others of SRBCT dataset

**Rank**	**Index no.**	**Clone ID**	**RPV (%)**
1	1916	80109	98.61
2	836	241412	98.24
3	783	767183	98.04
4	846	183337	98.02
5	1735	200814	97.81
6	1387	740604	97.40
7	335	1469292	96.35
8	1884	609663	96.16
9	1725	813630	95.69
10	1295	344134	95.48
14	2230	417226	94.45
17	1915	840942	94.22
19	1158	814526	93.24
25	85	700792	91.70

In the case of NB (Neuroblastoma) class, there were no exclusively discriminating genes reported in [35]. They were reported to be differentially expressed either with EWS or RMS. Among them, Genes 951, 1980, 2303 and 1626 have been identified as stronger genes pertaining to EWS by our method. Genes with index numbers 1764, 742, 236, 255, 417, 1601, 2199, 153, 1066, 2144, 2050 and 1662 were listed among 96 discriminating genes not pertaining to any class. Our method has allocated them to be in favour of NB class. A list of such genes selected for classifying NB versus others by original work as well as our method have been listed in Table 3.

**Table 3:** A list of features selected by using relative percentage variation of gene expression profiles between NB versus others of SRBCT dataset

**Rank**	**Index no.**	**Clone ID**	**RPV (%)**
1	1764	44563	96.29
2	742	812105	95.93
3	236	878280	95.38
4	255	325182	89.34
5	2202	110503	88.23
6	417	395708	85.49
7	909	785933	84.32
8	1601	629896	82
9	2199	135688	81.02
10	695	376516	80.50
18	2144	308231	69.75
25	2050	295985	60.40

The fourth class in SRBCT is RMS. Originally, 92 genes were listed in discriminating genes in [35]. All 92 genes have been selected in the list of strong genes by our method. But most of them reserve their slots in rank from 25 to 50. The genes with index 714, 2146, 1055, 554 and169 have come under the top 25 strongest genes list. Among them IGF2 (index 714) and MYL4 are specific for muscle tissues which have also been reported in RMS in [39,40]. Most of the top ranked genes in Table 3 are dominant in NB category in comparison with their expression in other classes (See additional_file 2). This implies that most of the top ranked genes in Table 3 are highly related to the classification of NB from other categories. Expression levels of these genes show that they can be classified using Golub's classification algorithm [13] since they appear to be highly correlated to the "idealized expression pattern" [13].

### Acute leukemia data

The experimental setup used for getting Acute leukemia data and other details can be found at [10]. The data set is publicly available at [42]. The microarray data consists of 7129 human genes and consists of 72 samples. The data is split into a training set and a test set. Training set consists of 38 samples comprising 27 AML samples and 11 AML samples. Test dataset of 34 samples comprising 20 ALL and 14 AML samples. As a test case, important genes to classify AML versus ALL are selected on the basis of their relative percentage variations of expression levels between two classes. Many genes reported in [10] are listed in Table 4 but in different order. Index number refers clone ID here. Among these selected genes, Genes with index numbers 2288, 1882, 6200 and 2043 have been reported as important genes in discovering AML class in the original work at [10]. Also, Genes with index numbers 5599, 2288, 5376 and 1882 have been reported to be important genes at [14] where genes were selected using mutual information.

**Table 4:** A list of features selected by earlier methods and using relative percentage variation of gene expression profiles of Golub within top 20 slots.

**Rank**	**Index No.**	**RPV %**
1	5599	99.99
5	1882	99.95
11	5376	99.91
12	6218	99.89
17	2288	99.81
19	2043	99.76
20	6200	99.75

Genes with index numbers 1882, 6218, 2288 and 6200 have been reported to be important genes selected using T-scores [14]. Gene 2242 has been reported as one of the important genes at [36]. Also, most of the other important genes reported to be important are found to occupy almost the first 50 genes in this method. This clearly shows that this method of feature selection is worthy one and may be used in conjunction with different methods of classification.

But, when a dataset with only two classes like the Golub data, selecting distinct genes do not workout since this method clearly bisects the genes into two distinct clusters one for each type. So, the number of important genes selected is relatively high in comparison with that for other datasets where the number of classes is more than two. For the datasets having more than two classes the feature selection method proposed here is found to be more useful.

### Breast cancer dataset

Next, we examined our proposed method of feature selection on hereditary breast cancer data from [29]. This dataset consists of twenty two breast tumor sample from 21 patients. Classification of each tumor sample into one of the classes based gene expression data was performed using a compound covariate predictor in [29]. In [36], the same classification was performed using SMC method and the genes were selected using a Gibb's sampler. The genes selected using our method to classify BRCA1 versus others is very close to those selected by Gibb's sampler in [36]. The genes with indices 10, 955, 2428, 2734,585, 1288 and 1620 have been selected among top 25 genes using our method (Table 5) but with little difference in order. Some other genes presented in [36] are found within top 50 genes selected in our method. Among all these genes gene with index number 10 is reported as very important for all the methods in [10,43]. It is observed in [36] that only with five or ten genes selected the classification was successful. This suggests that the genes selected by our method are worthwhile to use for classification of BRCA1 versus others since more of them are also found in the list mentioned in [36]. Gene 2272 has been identified as one of the top 20 strongest genes selected by mutual information [14]. Genes with index numbers 2734,2670,2893,1999 and 3009 which are also selected as the strongest genes in [14] are ranked between 26 and 45 by our method.

**Table 5:** A list of features selected by the original work within top 25 slots and using relative percentage variation of gene expression profiles between BRCA1 versus others.

**Rank no**	**Index No.**	**RPV %**
4	955	91.99
8	1288	90.30
15	585	88.22
16	2248	88.12
23	10	86.66
24	1620	86.41
25	2734	85.48

## Conclusion

In the present paper, we have treated the problem of feature selection of microarray gene expression data. We analyzed capability of the wavelet power spectrum using Haar wavelet in the domain of feature selection problem. We found that the power spectrum technique has the potential to identify the informative features. We proposed a clustering and feature selection method useful for classification based on Haar wavelet power spectrum. The top genes have been selected and have been compared with the results obtained in earlier works. In earlier works, preprocessing methods to remove noise or outliers before applying their methods were used. In the present work, to test the robustness of the dataset, no such preliminary measures were adopted. The method is quite simple in comparison to other feature selection methods and for implementation it needs no special software since the accessibility of wavelets is made quite easier in already available software. Each earlier works select different set of genes for classification purpose and proved only few genes are quite enough to approach the classification problem [14]. So, the present method can be used in conjunction with many established classification methods with lesser number of samples than that required for other methods. Many of the genes selected by our method have been used in the classification of earlier works which proves these genes are informative. The initial results of the idea of using Haar wavelet power spectrum in feature selection using microarray data are encouraging and due to its simplicity, speed and effectiveness and fitness for a wide range of datasets, it may be further researched for devising simpler tools with more optimization. A possibility of developing simpler but effective tools in this domain using wavelet power spectrum has been explored. Future research may be executed to utilize the power spectrum technique in the area of genomic signal processing using microarrays and its application.

## Method

In our approach of gene selection, we use the wavelet transforms of genes and the global spectral average of wavelet power spectrum over genes to select the genes useful for classification. The use of wavelet transforms provides economical and informative mathematical representations of many objects of interest [44]. Surveys of wavelet applications in biological data and in data mining are presented at [45-47]. The accessibility of wavelets has been made easier through many easily available software packages. Wavelet analysis is capable of providing analysis in a global fashion which is necessary in case of microarray data analysis.

**Figure 2:**
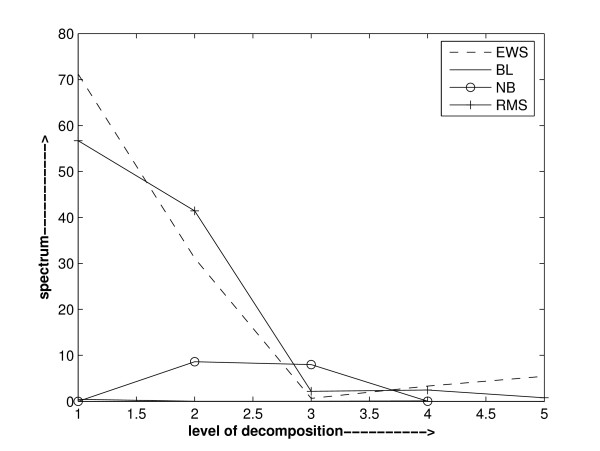
Haar wavelet power spectrum of gene 1 of SRBCT data in different diagnostic categories. It is obvious that the power spectrum of gene 1 is different in different diagnostic categories. Raw gene expression data of gene 1 is used in calculating the wavelet power spectrum.

**Figure 3:**
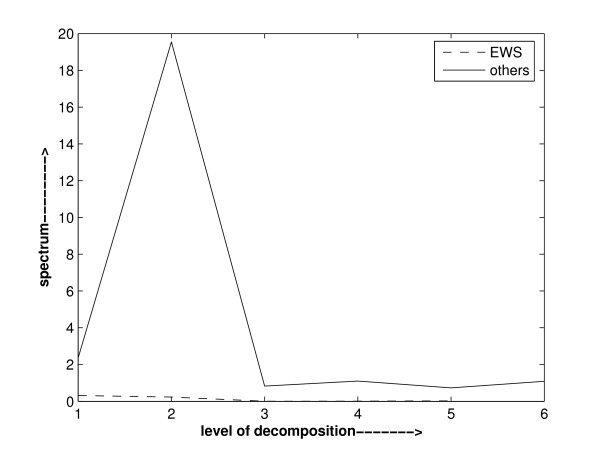
Haar wavelet power spectrum of gene 2 in EWS category data and that in the data containing all other categories of SRBCT data. It shows that gene2 is not dominant in EWS diagnostic category against the group of all other diagnostic categories. Raw gene expression data of gene 2 is used in calculating the wavelet power spectrum.

**Figure 4:**
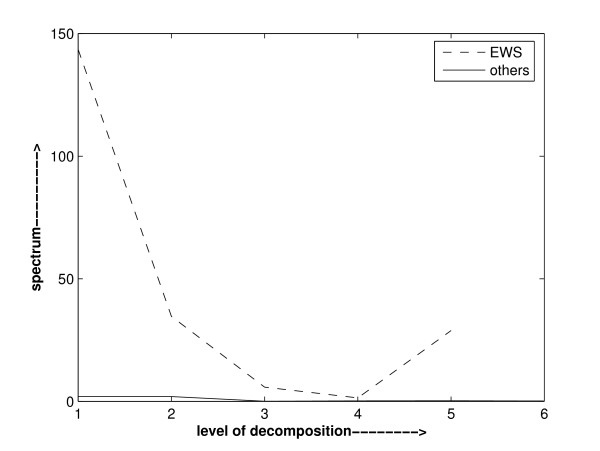
Haar wavelet power spectrum of gene 1319 in EWS category data and that in the data containing all the remaining categories of SRBCT data. Unlike gene2, gene 1319 is dominant in EWS against all other diagnostic categories. Raw gene expression data of gene 1319 is used in calculating the wavelet power spectrum.

The nature of genes, either being active, hyperactive or inactive, in different diagnostic categories is different and in varying amount. So, it may be observed while analyzing the wavelet power spectrum that it may not be same in all diagnostic categories. Based on this observation, a method to select important features relevant to each category against others can be devised. We analysed the power spectrum of various genes of different cancer datasets. It is observed that the power spectrum of a gene is not the same in all diagnostic categories. It is also observed that a gene is dominant in a particular diagnostic category against the group of the remaining categories. On the basis of these observations, we can device a method to pinpoint the important genes. These are illustrated in Figure 2, Figure 3 and Figure 4. Figure 2 depicts the power spectra of gene1 of SRBCT dataset [35] in various diagnostic categories. It is obvious from Figure 2 that the power spectrum of gene1 is not the same in all the diagnostic categories of SRBCT data. Power spectra found in Figure 3 depicts the spectra of the data of gene 2 in EWS diagnostic category and that of the data comprised of the remaining categories. It is obvious from Figure 3 that gene 2 is not dominant in EWS category. Instead it is dominant in the group of data comprised of the remaining categories. Figure 4 depicts the similar power spectra of another gene 1319. It also contains two spectra: one created using the data of gene 1319 in EWS category and the other created using the data of gene 1319 comprised of the remaining categories. A comparison of power spectra of gene 2 and gene 1319 reflects alternative trend present in genes.

On the basis of the trends observed, we defined a merit called relative percentage variation (RPV) to select the genes useful for distinguishing a diagnostic category from others. The dataset was divided into two subsets before performing further computation. One subset contained only the samples of a particular diagnostic category for which features are to be selected. The other subset was comprised of the samples of the remaining all other categories. Global averages of the wavelet power spectra over genes in these two subsets of a data were calculated.

The relative percentage variation (RPV) of the global average spectra of the genes against that of the other subset is calculated using the formula RPV=(x1−y1)x1×100%
 MathType@MTEF@5@5@+=feaafiart1ev1aaatCvAUfKttLearuWrP9MDH5MBPbIqV92AaeXatLxBI9gBaebbnrfifHhDYfgasaacH8akY=wiFfYdH8Gipec8Eeeu0xXdbba9frFj0=OqFfea0dXdd9vqai=hGuQ8kuc9pgc9s8qqaq=dirpe0xb9q8qiLsFr0=vr0=vr0dc8meaabaqaciaacaGaaeqabaqabeGadaaakeaacqqGsbGucqqGqbaucqqGwbGvcqGH9aqpdaWcaaqaaiabcIcaOiabbIha4naaBaaaleaacqaIXaqmaeqaaOGaeyOeI0IaeeyEaK3aaSbaaSqaaiabigdaXaqabaGccqGGPaqkaeaacqqG4baEdaWgaaWcbaGaeGymaedabeaaaaGccqGHxdaTcqaIXaqmcqaIWaamcqaIWaamcqGGLaqjaaa@4174@ where x_1 _and y_1 _are the global averages of genes in a particular diagnostic category and in the second subset. This clearly divided the data into two clusters. One cluster contained the genes with positive RPV. The other cluster contained the genes with negative RPV. Cluster with genes with positive RPV was selected as favourable for classification. If a gene has an average expression higher in the particular diagnostic category than that in the second subset, it will have a positive RPV. The genes in the selected cluster were ranked according to their RPVs. The same procedure was followed to select the dominant genes for other diagnostic categories.

Standard datasets used in already established works [13,1,36,35] were used to check the validity of the proposed method. The genes selected for these standard datasets were observed to be in tune with those reported in earlier works [13,1,36,35]. Also, the present method is simpler than the methods used in the earlier works [13,1,36,35]. Thus, the results obtained by our method are encouraging in both clustering genes and feature selection in the context of classification and hence found useful. A possible indication to the use of wavelet power spectrum in the feature selection domain to develop more simple methods is imminent from our work and further research may be continued to find more strategies in this domain using wavelet power spectrum.

## Authors' contributions

PS conceived the idea, implemented and tested and drafted the manuscript. RS and SV have been involved in revising the manuscript for important intellectual content. SV took part in testing the design along with SP. All the authors have read and approved the final manuscript.

## Supplementary Material

Additional File 1 Expression levels of the top 10 genes selected as informative for classifying BL category against the other classes. Most of the genes selected as informative are dominant in BL category (from sample order 24–31 along x-axis). It shows that most of the selected genes are highly related for classification of BL category. Click here for file

Additional File 2 Expression levels of the top 10 genes selected as informative for classifying NB category against the other classes. Most of the genes selected as informative are dominant in NB category (from sample order 32–43 along x-axis). It shows that most of the selected genes are highly related for classification of NB category. Click here for file
